# Double Plating With Fibular Allograft Reinforcement of Scapular Spine Fracture After Reverse Shoulder Arthroplasty

**DOI:** 10.1016/j.eats.2024.103050

**Published:** 2024-06-17

**Authors:** Alexandre Lädermann, Jeanni Zbinden, Alaa Elsenbsy, Sumanth Nayak, Alberto Guizzi, Philippe Collin

**Affiliations:** a Division of Orthopaedics and Trauma Surgery, La Tour Hospital, Meyrin, Switzerland; b Faculty of Medicine, University of Geneva, Geneva, Switzerland; c Division of Orthopaedics and Trauma Surgery, Department of Surgery, Geneva University Hospitals, Geneva, Switzerland; d Department of Orthopedic and Trauma Surgery, Faculty of Medicine, South Valley University, Qena, Egypt; e Tejasvini Hospital and SSIOT, Mangaluru, India; f Department of Medical and Surgical Specialties, Radiological Sciences, and Public Health, University of Brescia, Brescia, Italy; g CHP Saint-Gregoire, Saint-Grégoire, France; h Clinique Victor Hugo, Paris, France; i American Hospital of Paris, Neuilly-sur-Seine, France

## Abstract

Scapular spine fractures following reverse shoulder arthroplasty have been associated with complications that include nonunion and fixation failure. This Technical Note presents a surgical approach for enhancing the stability and strength of spine fracture osteosynthesis. The method involves the utilization of double plating in conjunction with fibular allograft reinforcement anchored in the supraspinous fossa to provide support under the acromion. The allograft, offering an enhanced structural integrity, may contribute to an improved rate of bone fusion and clinical outcomes without donor site morbidity.

The altered biomechanics associated with reverse shoulder arthroplasty (RSA), characterized by heightened deltoid forces, has led to a surge in traumatic or fatigue (stress) fractures of the scapular spine and acromion.^1^^,^^2^ These fractures pose a challenge to both diagnosis and treatment, with growing debate between conservative and operative approaches.^1^^,^^3^, ^4^, ^5^, ^6^

Open reduction and internal fixation (ORIF) of fractures associated with RSA presents a high complication rate and inconsistent results.^7^ These include nonunion and fixation failure due to continuous strain by the deltoid along the bony fragments. Fixation failure may result from insufficient stability of the osteosynthesis, particularly in osteoporotic bone. There is consequently a pressing need for innovative techniques that can enhance fixation stability, especially in patients with compromised bone quality.^7^

This Technical Note aims to describe a surgical approach involving double plating associated with fibular allograft reinforcement anchored in the supraspinous fossa and supporting the undersurface of the acromion.

## Surgical Technique

### Preoperative Decision-Making

**Fig 1:**
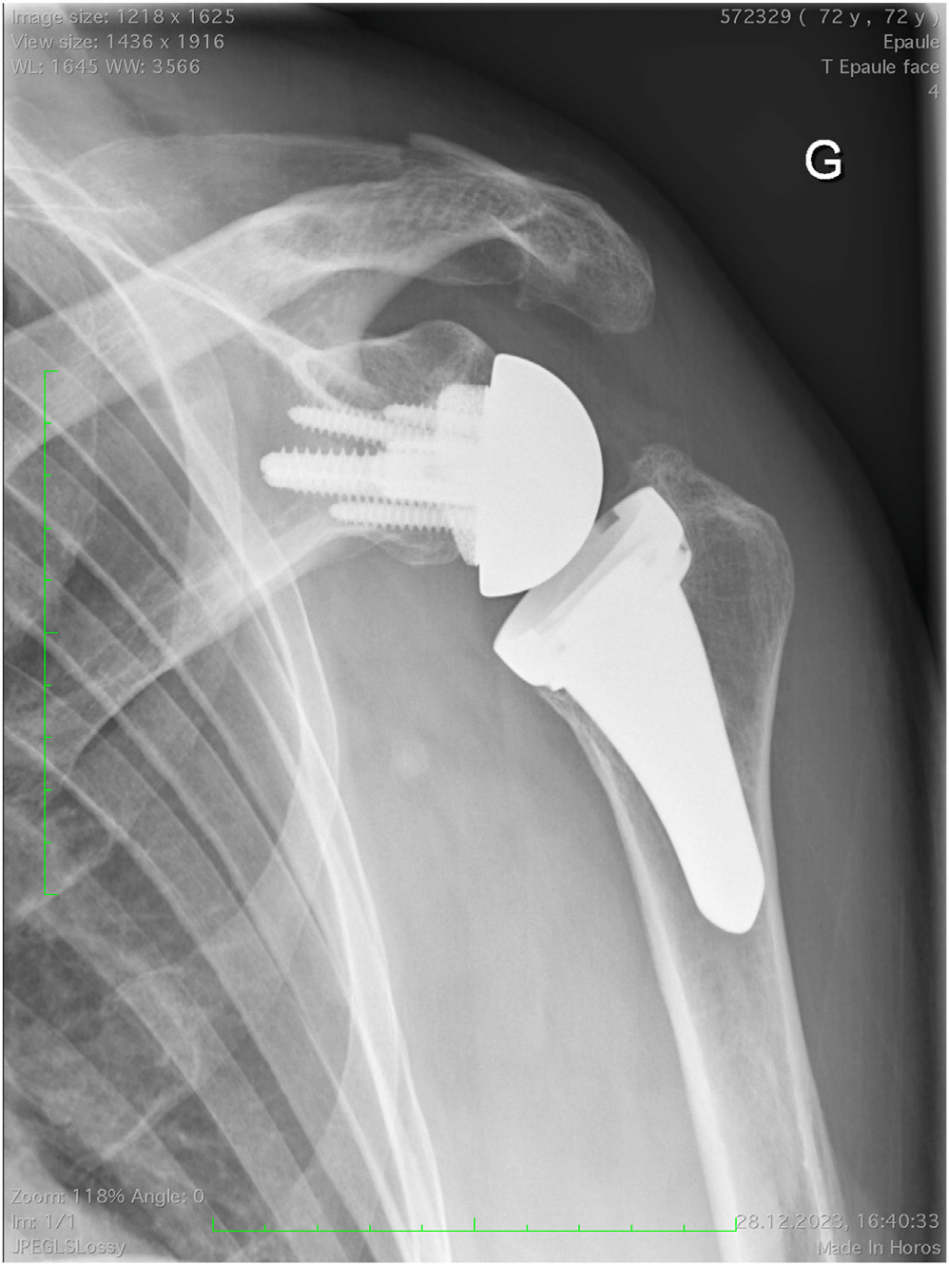
Anteroposterior x-ray of a left shoulder. A reverse shoulder arthroplasty had been implanted 3 years previously with satisfactory results according to the patient. He sustained an ipsilateral traumatic scapular spine fracture in a skiing accident.

**Fig 2:**
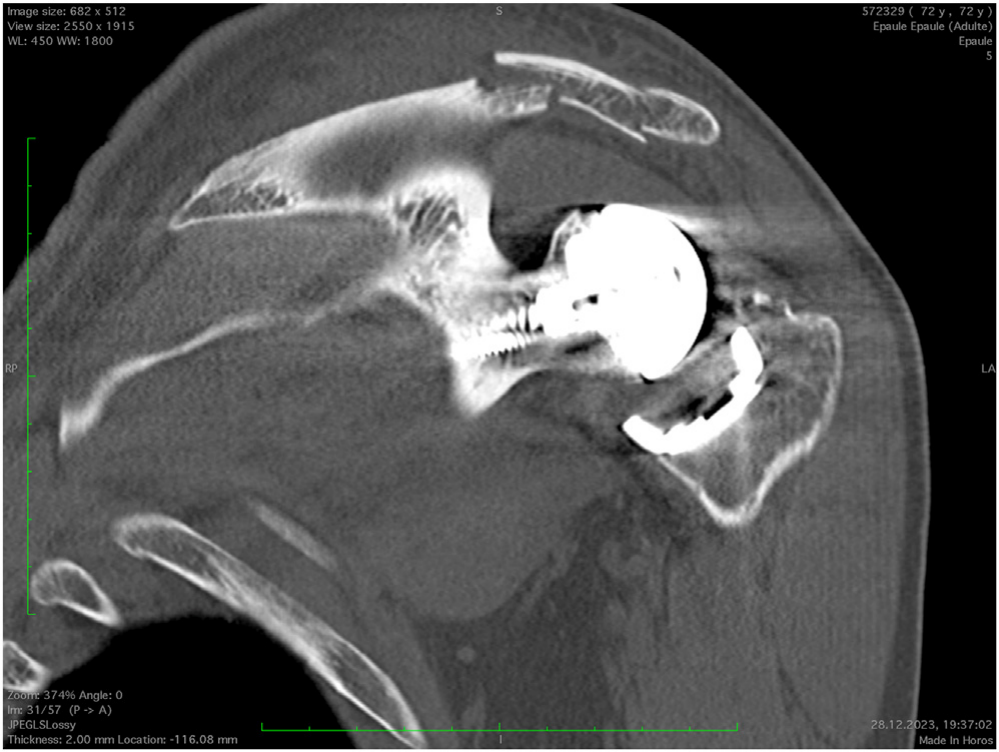
Coronal view of a left shoulder on computed tomography. Observe the displaced bifocal fracture of the scapular spine and the resulting inferior tilt of the acromion.

Causes of acromion and scapular spine fractures after RSA are categorized into either acute traumatic or fatigue fractures. Displaced traumatic fractures tend to be treated operatively, whereas conservative treatment is favored for the non- or minimally displaced fatigue fractures. Indications and contraindications are listed in Table 1. All patients undergo careful clinical and appropriate radiographic examination, including standard radiography (Fig 1) and computed tomography (Fig 2). Considering the severely painful nature of this condition, ORIF is performed as soon as possible if surgical intervention is deemed necessary.

**Table 1:** Indications and Contraindications for Scapular Spine Fractures or Nonunion

Indications	Relative Contraindication	Contraindications
Displaced and symptomatic traumatic fractures impacting a significant portion of the deltoid	Displaced fatigue fractures of the scapular spine	Nondisplaced fractures
Symptomatic nonunion		Patient refusal
		Active prosthetic joint infection

### Patient Setup

The patient is positioned at a seated angle of 60° and tilted approximately 20° to the opposite side on a specialized shoulder operating table. The scapula on the affected side is draped freely. The left arm is secured in an arm holder designed for shoulder surgery to facilitate fracture reduction (Video 1).

**Fig 3:**
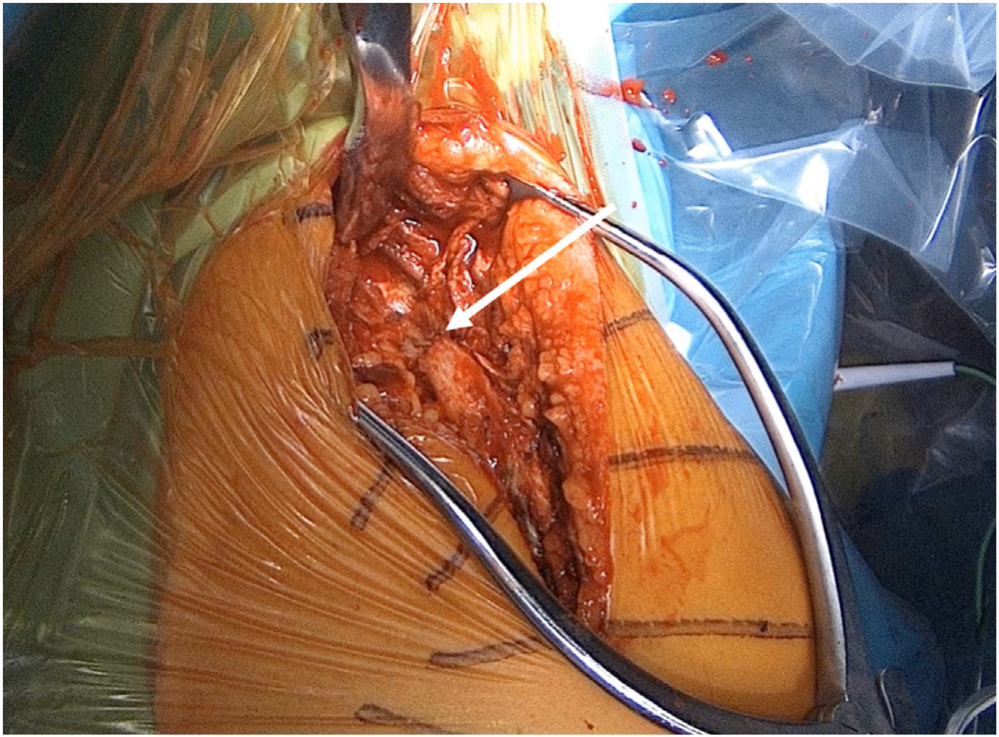
Lateral view of a left shoulder. Gelpi and Hohmann retractors expose a scapular spine fracture (white arrow).

A posterior, longitudinal, mediolateral incision is made along the scapular spine and curved along the acromion, ensuring minimal unnecessary bone stripping. The fracture is identified (Fig 3).

**Fig 4:**
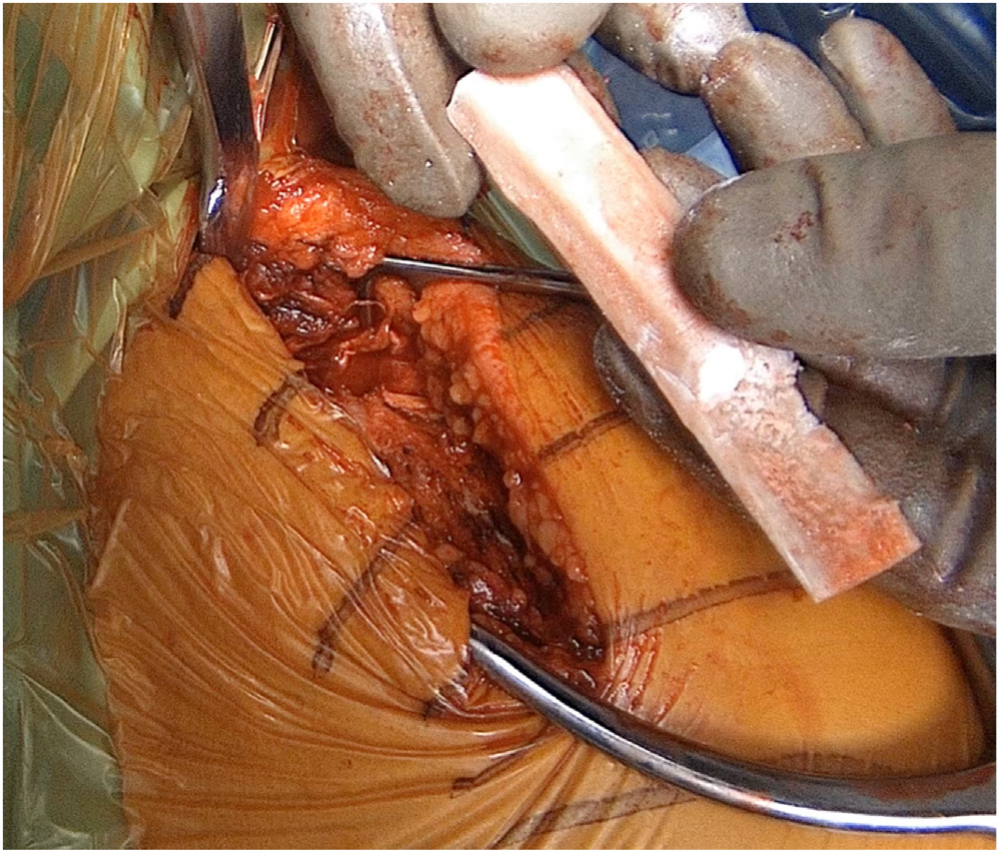
Lateral view of a left shoulder demonstrating the undersurface of a reshaped allograft. Note the graft, which was bezeled/cut at an angle to provide a thin support under the acromion.

**Fig 5:**
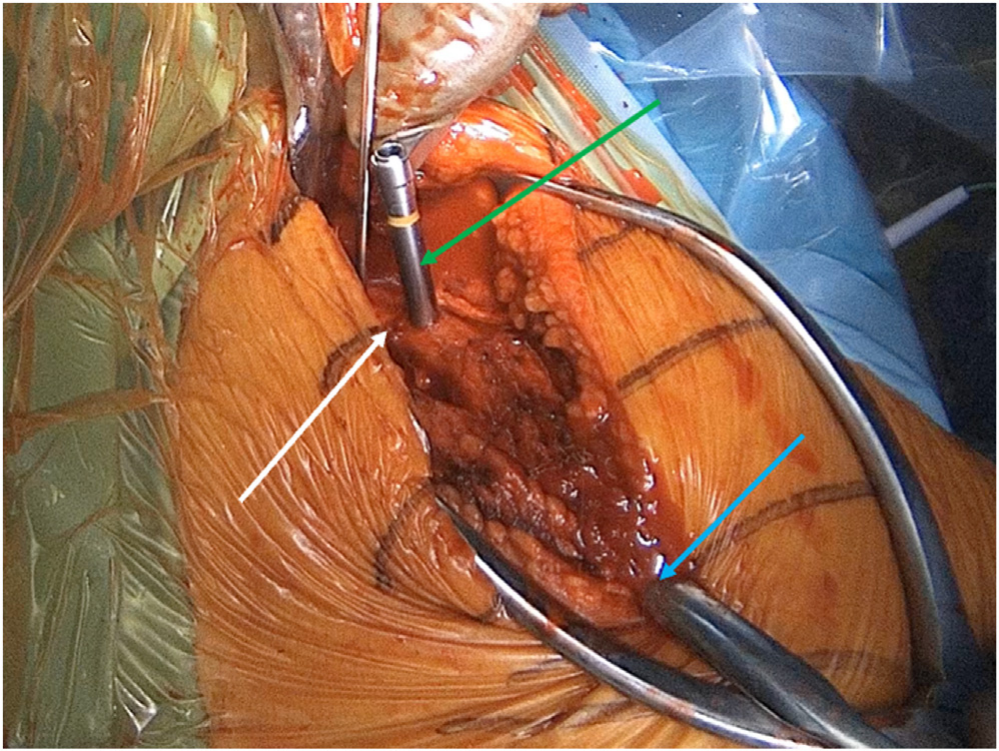
Lateral view of a left shoulder illustrating the maneuver used to obtain fracture reduction. The scapular spine fracture (white arrow) is reduced by applying direct pressure on the lateral fragment (green arrow) and laterally introducing a thin Hohmann retractor while pushing the lateral acromion superiorly (blue arrow). Meanwhile, the shoulder is maintained in abduction within the arm holder.

Next, a fibular allograft is carefully modeled to provide a thin support under the acromion that does not impede free range of motion (Fig 4, Video 1). The graft is introduced into the supraspinous fossa by sliding it medially to laterally. Its position beneath the acromion is secured using an 8-mm thin Hohmann retractor passed laterally through the deltoid. Additionally, a temporary Kirchner wire is used to stabilize the graft (Video 1). The fracture is anatomically reduced through a maneuver including direct pressure on the lateral fragment; a thin Hohmann introduced laterally, pushing the acromion superiorly; and abduction of the shoulder performed with the arm holder (Fig 5). Temporary Kirchner wires are utilized to maintain this reduction (Video 1).

**Fig 6:**
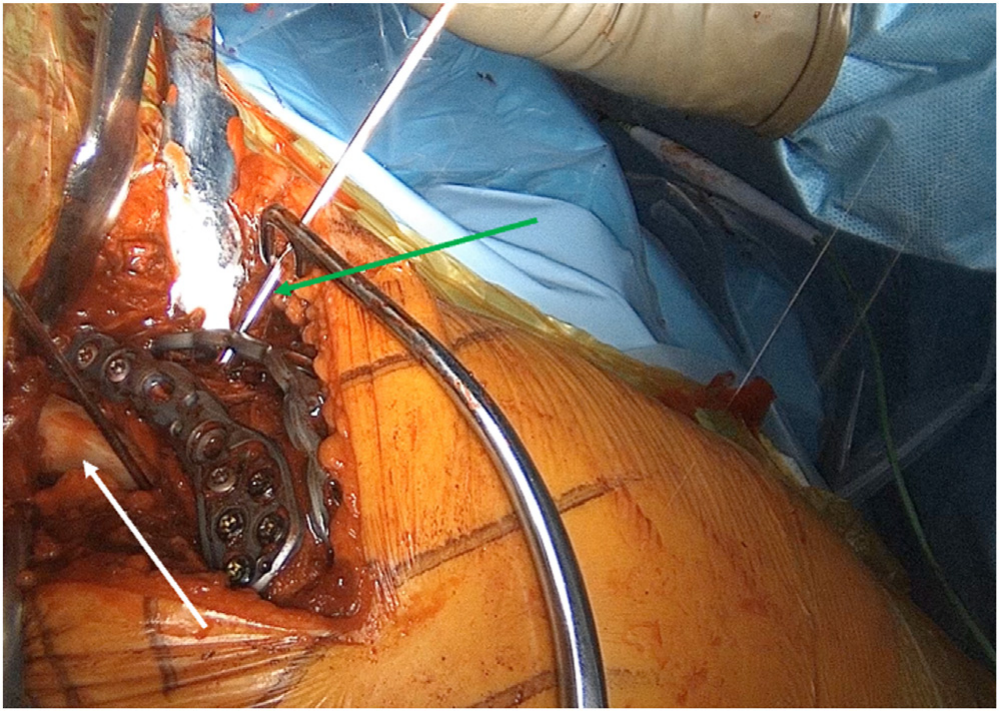
Lateral view of a left shoulder, demonstrating the final construction achieved using 90° double plating. The drill (green arrow) and the screws must reach the allograft (white arrow) lying in the supraspinatus fossa.

A 3.5 lateral clavicular plate is bent to match the contours of the acromion and scapular spine. Slight overcorrection is desired. The plate is then applied to the superior aspect of the scapular spine. Initially, it is fixed with lateral locking screws and subsequently with a combination of compression and medially placed locking screws. Some of the superoinferior screws in the acromion should reach the underlying graft. To create a double-plating construct at 90°, as described by Rouleau and Gaudelli,^8^ a second 3.5-mm plate is applied dorsally along the subcutaneous border of the scapular spine (Fig 6, Video 1). Compression screws are used to reach the acromion or the spine and, anteriorly, the graft (Fig 6).

**Fig 7:**
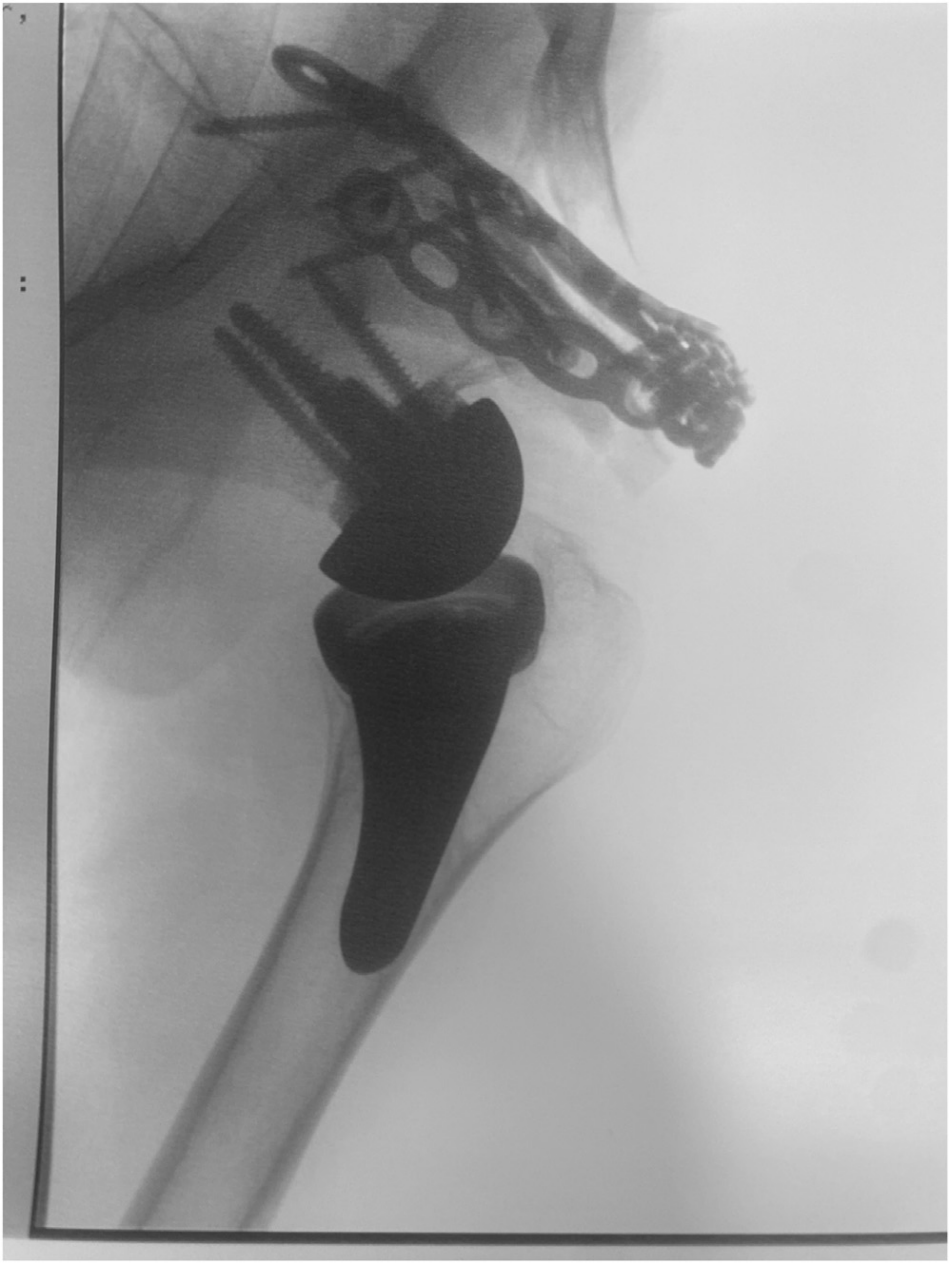
Anteroposterior fluoroscopic view of a left shoulder confirming satisfactory reduction of the fracture with a nonobstructed subacromial space.

Throughout the procedure, reduction of the fracture is verified by fluoroscopy (Fig 7). The subacromial space must remain unobstructed to allow unrestricted range of motion, and the prosthetic implant must remain congruent. The pearls and pitfalls of each surgical step and the advantages and disadvantages are summarized in Tables 2 and 3, respectively.

**Table 2:** Pearls and Pitfalls of Each Surgical Step

Surgical Steps	Pearls	Pitfalls
Patient setup	Arm holder is used to achieve tension-free reduction of the fracture.	Cost and encumbrance
Allograft reshaping	The lateral extremity is refined using a saw or ball mill cutter.	Risk of fracture of the allograft. However, fibular allografts are long enough to prepare 2 grafts.
Allograft introduction	Graft is introduced into the supraspinous fossa by sliding it medially to laterally.	Sliding of the graft laterally to medially risks damage to the deltoid.
Allograft stabilization	The graft is maintained under the acromion by a thin Hohmann retractor pushing the allograft superiorly. Additional Kirschner wire is used to improve stability temporarily.	Damage of the deltoid with the retractor.
Double-plating construct at 90°	Double plating provides significantly higher failure loads compared to single plating.^11^	A large number of screws/drilling may be a stress factor for secondary fractures or may prevent subsequent hardware removal.
Aftercare	Orthosis avoiding lower abduction angles for 4 to 6 weeks may be proposed depending on the solidity of the construct and the quality of the bone.	Patient compliance with a long period of immobilization.^15^

**Table 3:** Advantages and Disadvantages

Advantages	Disadvantages
Nonprotuberant allograft	Cost of the allograft
Bone stock restoration	No clinical data thus far
No loss of time due to harvesting	Additional procedure with prolonged operative
No donor site morbidity	
Conservation of prosthesis range of motion	
Inexpensive, readily available plates	
Respect of the deltoid muscle	

**Fig 8:**
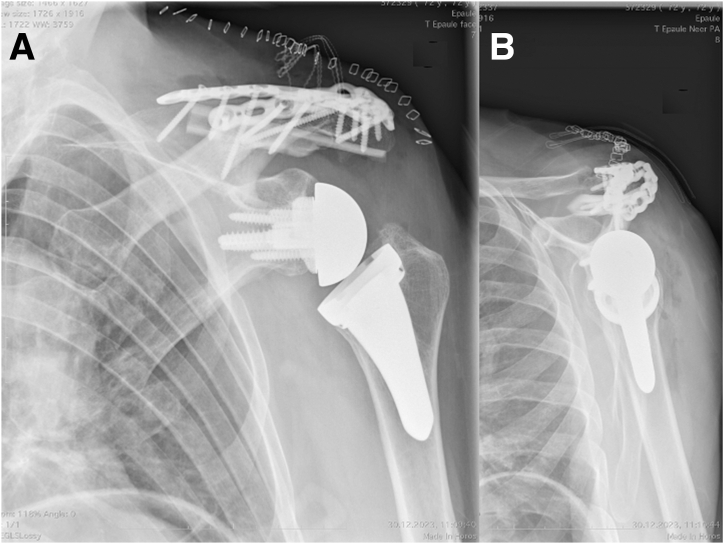
(A) Postoperative anteroposterior and (B) Neer views of a left shoulder demonstrating 90° double plating and fibular allograft positioning.

**Fig 9:**
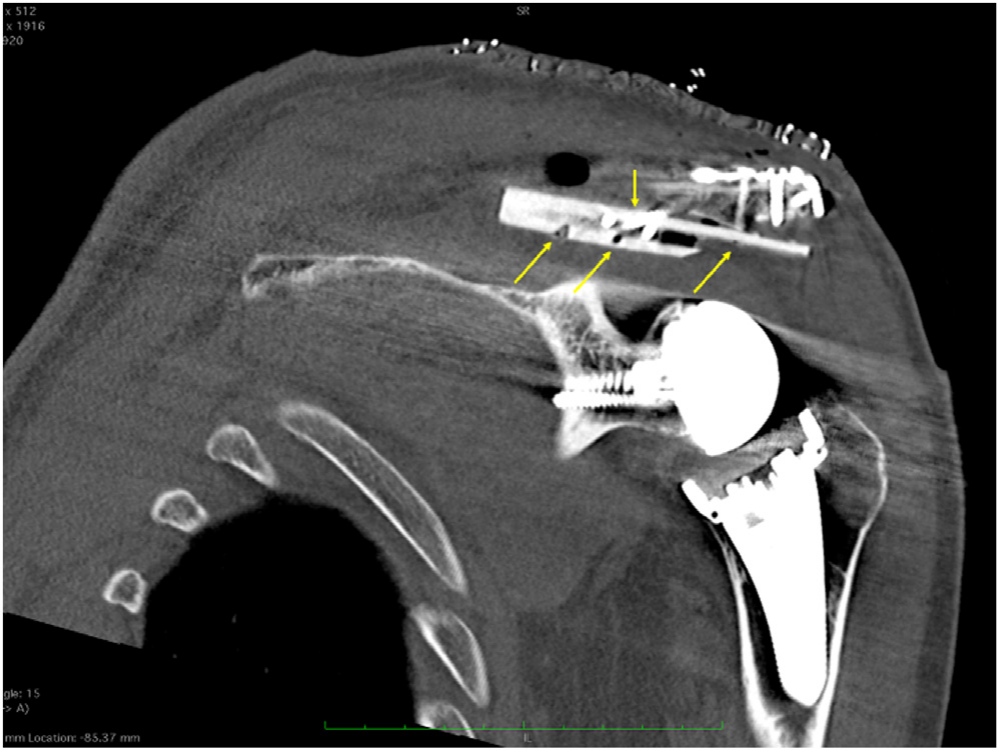
Coronal view of a left shoulder on computed tomography. The fibular allograft, fixed with screws (yellow arrows), offers support under the acromion.

**Fig 10:**
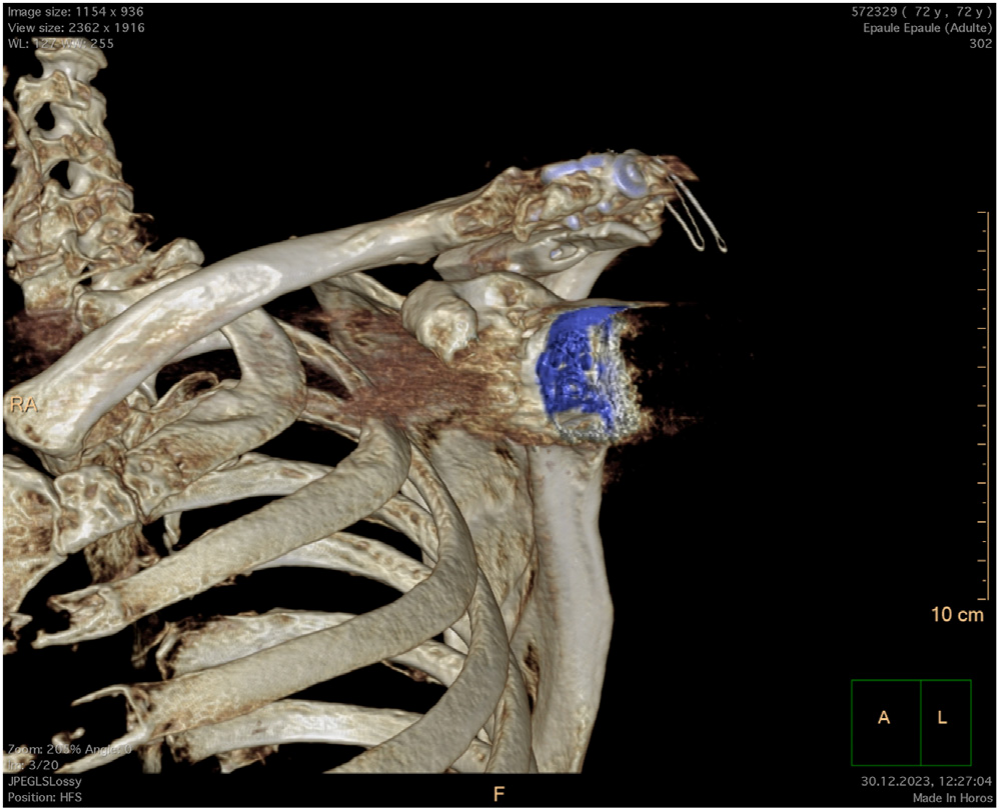
Postoperative 3-dimensional computed tomography reconstruction of a left shoulder with glenosphere and humerus subtraction. Note the fibular allograft reinforcement anchored in the supraspinous fossa to support the undersurface of the acromion.

Finally, after lavage, the trapezius and the deltoid muscles are closed above the plates. Postoperative images confirm satisfactory reduction of the fracture (Fig 8, Fig 9 to 10, Video 1).

### Postoperative Protocol and Rehabilitation

Anti-inflammatory medication is tolerated in the immediate postoperative period but is subsequently discontinued, as long-term use may compromise bone healing and allograft integration.^9^ Similarly, smoking should be avoided postoperatively to avoid adverse effects on bone and wound healing.^10^ Patients are immobilized in a simple sling or a 45° abduction pillow, depending on the solidity of the construct and the quality of the bone, for a period of 4 to 6 weeks. After 6 weeks, free active-assisted range is allowed. Muscle reinforcement, heavy lifting, and athletic activities involving the upper extremities are contraindicated for 3 months.

## Discussion

**Fig 11:**
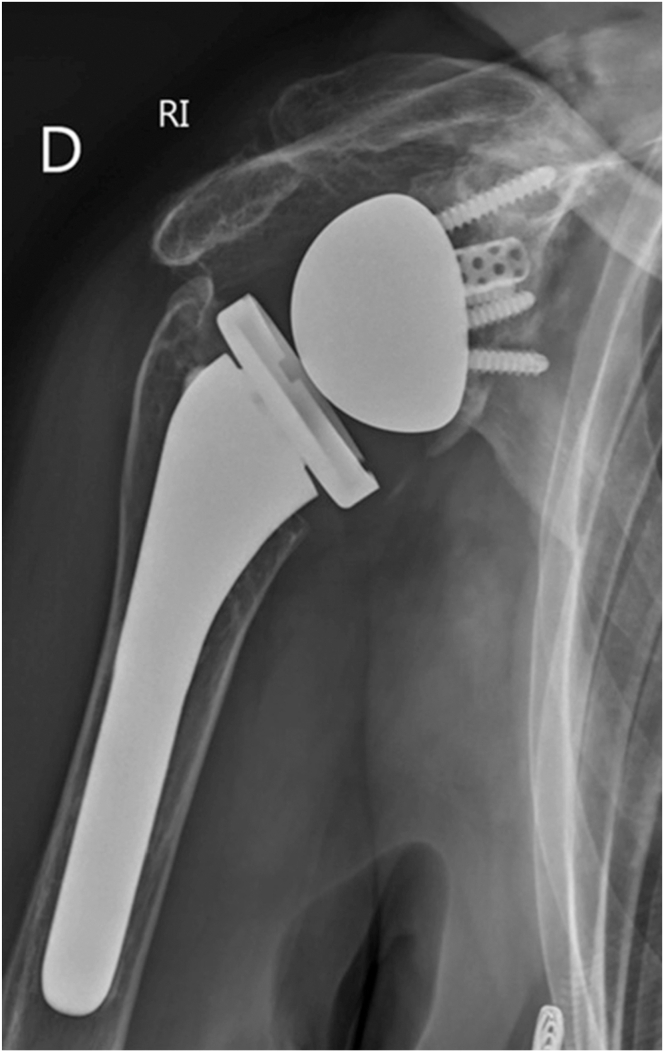
Right anteroposterior x-ray of a reverse shoulder arthroplasty complicated by a scapular spine fracture. The inferior tilt has led to secondary impingement.

Displacement of scapular spine fractures can result in severe pain, frequent secondary displacement, and impingement due to the inferior tilt of the weakened distal fragment and reduced deltoid muscle strength (Fig 11). This affects the function and mobility of the arm, especially in patients who have undergone RSA. Reluctance to address these fractures is due primarily to the difficulty of achieving a satisfactory reduction and stable fixation required for bone healing. Previous research has emphasized the need to enhance initial stability, especially in osteoporotic bone.^7^ The present study describes an elegant solution that involves double plating of scapular spine fractures, which is known to provide significantly higher failure loads compared with single plating.^11^ This approach appears to be enhanced biomechanically by a long and robust allograft that provides support under the acromion, further encouraging bony fusion. Nonetheless, the authors emphasize the need for further research to biomechanically prove resistance of the allograft in initial stability to significant loads from contraction of the deltoid. Long-term clinical results must also be confirmed.

Advantages of the proposed solution are numerous. First, the allograft, concealed within the anterior portion of the supraspinatus fossa, does not protrude and should not cause discomfort. Second, the fibular graft restores bone stock of thin and osteoporotic acromion.^12^ Third, the allograft is laterally reshaped to prevent subacromial abutment impingements (Fig 4),^13^ without compromising subsequent prosthesis range of motion. Fourth, the hard allograft significantly enhances the fixation of the screws that penetrate. Fifth, the allograft avoids potential donor site morbidity. Finally, our proposal uses standard, inexpensive plates available in all trauma facilities and respects the deltoid muscle, in contrast to other dedicated plates for acromial fractures that have been proposed.^14^ Such a specific device seems to be rarely used and risk damage to the deltoid muscle with hooks. The main inconvenience of our proposal is the associated costs of the allograft.

The proposed technique employs double plating in conjunction with fibular allograft reinforcement anchored in the supraspinous fossa for scapular spine fractures after RSA. The allograft, providing support under the acromion and enhancing structural integrity, may contribute to an improved rate of bone fusion and clinical outcomes without donor site morbidity.

## Disclosures

The authors report the following potential conflicts of interest or sources of funding: Supported by FORE (Foundation for Research and Teaching in Orthopaedics, Sports Medicine, Trauma, and Imaging in the Musculoskeletal System) (Grant FORE 2023-77). Investigation performed at FORE (Foundation for Research and Teaching in Orthopedics, Sports Medicine, Trauma, and Imaging in the Musculoskeletal System). A.L. is a paid consultant for Arthrex, Stryker, Medacta, and Enovis; has received royalties from Stryker and Medacta; is the (co)founder of FORE, Med4Cast, and BeeMed; owns stock options in Medacta and Follow Health; and is on the board of the French Arthroscopic Society. P.C. receives royalties from and is a consultant and paid speaker for Stryker and Enovis, is the cofounder of Med4Cast and Follow, and is on the board of SECEC and IBSES. J.Z., A.E., S.N., A.G. and P.C. declare that they have no known competing financial interests or personal relationships that could have appeared to influence the work reported in this paper. Full ICMJE author disclosure forms are available for this article online, as supplementary material.

## Supplementary Data

ICMJE author disclosure forms

Video 1 Demonstration of a technique that involves double plating of a scapular spine fracture with fibular allograft reinforcement anchored in the supraspinous fossa to support the undersurface of the acromion after reverse shoulder arthroplasty (RSA). This case illustrates a 72-year-old patient who had successfully undergone RSA 3 years earlier. He sustained an ipsilateral traumatic scapular spine fracture during skiing. The patient is positioned in a beach-chair position with the left arm secured in an arm holder to facilitate fracture reduction. A posterior, longitudinal, mediolateral incision is made along the scapular spine and the fracture is identified. A fibular allograft is carefully modeled to provide a thin support under the acromion. The graft is introduced into the supraspinous fossa by sliding it medially to laterally. A temporary Kirchner wire is used to stabilize the graft. The scapular spine fracture is reduced by applying direct pressure on the lateral fragment and laterally introducing a thin Hohmann retractor while pushing the lateral acromion superiorly. Meanwhile, the shoulder is maintained in abduction within the arm holder. The final construction is achieved using 90° double plating, stabilizing at the same time the fibular allograft lying anteriorly in the supraspinatus fossa. X-rays and 2-dimensional and 3-dimensional computed tomography scan reconstruction confirmed a satisfactory reduction of the fracture and a stable construct.
